# Hirshfeld surface analysis and crystal structure of 7-meth­oxy-5-methyl-2-phenyl-11,12-di­hydro-5,11-methano-1,2,4-triazolo[1,5-*c*][1,3,5]benzoxadiazo­cine

**DOI:** 10.1107/S2056989018010848

**Published:** 2018-08-10

**Authors:** Mustafa Kemal Gumus, Sevgi Kansiz, Necmi Dege, Valentina A. Kalibabchuk

**Affiliations:** a Artvin Coruh University, Science-Technology Research and Application Center, Artvin 08000, Turkey; b Ondokuz Mayıs University, Faculty of Arts and Sciences, Department of Physics, 55139, Kurupelit, Samsun, Turkey; c Department of General Chemistry, O. O. Bohomolets National Medical University, Shevchenko Blvd. 13, 01601 Kiev, Ukraine

**Keywords:** crystal structure, Biginelli condensation, benzoxa­diazo­cine, Hirshfeld surface

## Abstract

The title compound, C_19_H_18_N_4_O_2_, crystallizes with two independent mol­ecules in the asymmetric unit. The triazole ring is inclined to the benzene rings by 9.63 (13) and 87.37 (12)° in one mol­ecule, and by 4.46 (13) and 86.15 (11)° in the other. In the crystal, classical N—H⋯N hydrogen bonds, weak C—H⋯O hydrogen bonds and weak C—H⋯π inter­actions link the mol­ecules into a three-dimensional supra­molecular network. Hirshfeld surface analysis and two-dimensional fingerprint plots were used to investigate the inter­molecular inter­actions present in the crystal, indicating that the most important contributions for the crystal packing are from H⋯H (51.4%), H⋯C/C⋯H (26.7%), H⋯O/O⋯H (8.9%) and H⋯N/N⋯H (8%) inter­actions.

## Abstract 2

In the crystal, classical N—H⋯N hydrogen bonds, weak C—H⋯O hydrogen bonds and weak C—H⋯π inter­actions link the mol­ecules into a three-dimensional supra­molecular network.

## Chemical context   

One of the earliest known multi-component reactions (MCRs) is the Biginelli multi-component cyclo­condensation. Its variations are still a timely subject for research because of the near unlimited scope of this approach and the constant demand for mol­ecular diversity of small mol­ecules in many areas such as drug discovery, combinatorial and medicinal chemistry (Kappe, 2000 ▸; Slobbe *et al.*, 2012 ▸). As we had previously synthesized a type of oxygen-bridged Biginelli compounds derivatives, (Gümüş *et al.*, 2017 ▸), we decided to examine the structure of this heterocyclic system by X-ray analysis (Aydemir *et al.*, 2018 ▸; Gümüş *et al.*, 2018 ▸). In this study, a novel Biginelli-like assembly of 3-amino-5-(phen­yl)-1,2,4-triazole with acetone and 2-hy­droxy-3-meth­oxy­benzaldehyde has been developed to offer easy access to 7-meth­oxy-5-methyl-2-(phen­yl)-11,12-di­hydro-5,11-methano[1,2,4]triazolo[1,5-*c*][1,3,5]benzoxa­diazo­cine compounds as examples of a new class of heterocycles.
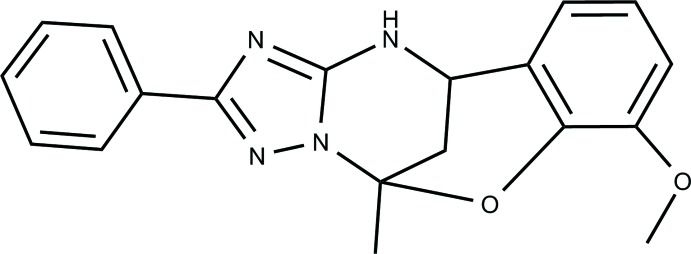


## Structural commentary   

**Figure 1:**
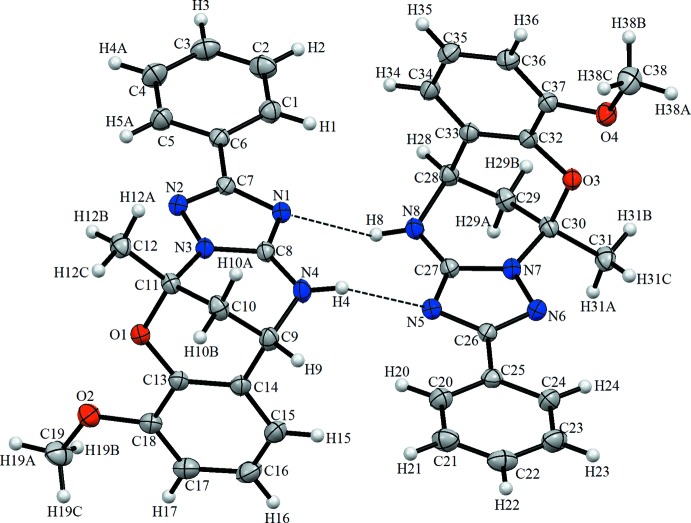
The mol­ecular structure of the title compound, showing the atom labelling. Displacement ellipsoids are drawn at the 20% probability level.

The asymmetric unit of the compound contains two independent mol­ecules (Fig. 1 ▸), linked by N4—H4⋯N5 and N8—H8⋯N1 hydrogen bonds, which stabilize the mol­ecular structure (Table 1 ▸, Fig. 1 ▸ and 2 ▸). The C11—O1, C13—O1, C18—O2, C19—O2, C30—O3, C32—O3, C37—O4 and C38—O4 bond lengths are all in agreement with single-bond character. The C—O bond distances observed are lower than in the literature [1.364 (4), 1.390 (4), 1.428 (4) and 1.443 (4) Å; Aydemir *et al.*, 2018 ▸). The triazole ring is inclined to the benzene rings by 9.63 (13) and 87.37 (12)° in one mol­ecule, and by 4.46 (13) and 86.15 (11)° in the other. The ring N3/C8/N4/C9–C11 is inclined to the ring N2/C7/N1/C8/N3 by 5.80 (14)° and to the ring C9–C11/O1/C13/C14 by 86.9 (6)° [equivalent values of 6.55 (11) and 85.29 (11)°, respectively, in the other independent mol­ecule].

## Supra­molecular features   

**Figure 2:**
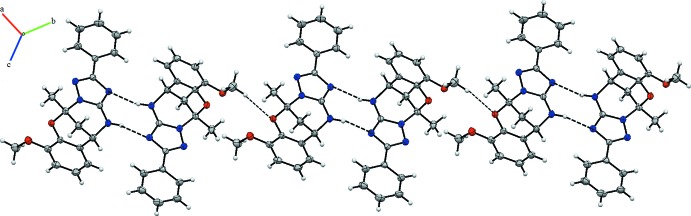
The view of the crystal packing of C_19_H_18_N_4_O_2_. Dashed lines denote the N—H⋯N and C—H⋯O hydrogen bonds.

In the crystal, weak C—H⋯O interactions link the pairs of independent molecules into layers parallel to (100) (Table 1 ▸; Fig. 2 ▸). The layers are further connected by weak C—H⋯π inter­actions, generating a three-dimensional supra­molecular structure.

## Hirshfeld surface analysis   

**Figure 3:**
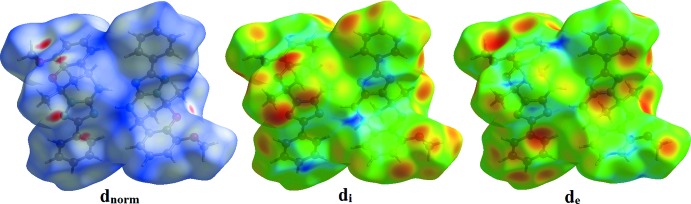
Hirshfeld surfaces of the title compound mapped over *d*_norm_, *d*_i_ and *d*_e_.

Hirshfield surface analysis was performed using *CrystalExplorer* (Turner *et al.*, 2017 ▸) to qu­antify the various inter­molecular inter­actions in the synthesized complex. The Hirshfeld surfaces of the title compound mapped over *d*_norm_, *d*_i_ and *d*_e_ are illustrated in Fig. 3 ▸. The red spots on the surface indicate the intermolecular contacts involved in strong hydrogen bonding and interatomic contacts (Sen *et al.*, 2018 ▸) and correspond to C—H⋯O hydrogen bonds in the title compound (Figs. 3 ▸ and 4 ▸). The Hirshfeld surfaces were calculated using a standard (high) surface resolution with the three-dimensional *d_norm_* surfaces mapped over a fixed colour scale of −0.249 (red) to 1.531 (blue) a.u..

**Figure 5:**
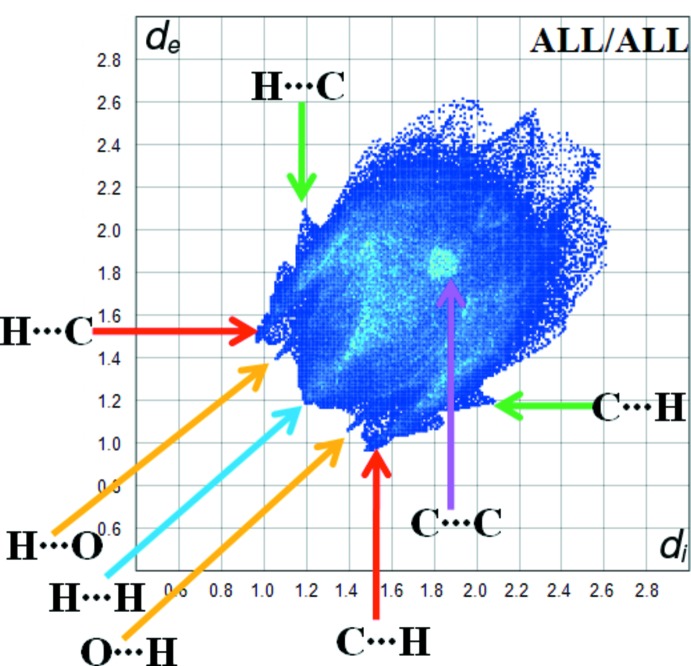
Fingerprint plot for the title compound.

**Figure 6:**
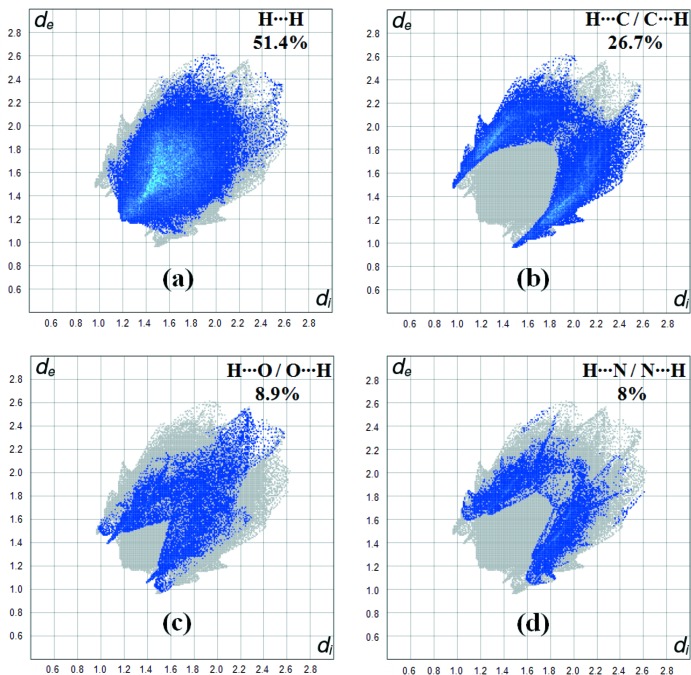
Two-dimensional fingerprint plots with a *d*_norm_ view of the (*a*) H⋯H (51.4%), (*b*) H⋯C/C⋯H (26.7%), (*c*) H⋯O/O⋯H (8.9%) and (*d)* H⋯N/N⋯H (8%) contacts in the title compound.

Fig. 5 ▸ shows the two-dimensional fingerprint of the sum of the contacts contributing to the Hirshfeld surface represented in normal mode. The graph shown in Fig. 6 ▸ *a* (H⋯H) shows the two-dimensional fingerprint of the (*d*_i_, *d*_e_) points associated with hydrogen atoms. It is characterized by an end point that points to the origin and corresponds to *d*_i_ = *d*_e_ = 1.2 Å, which indicates the presence of the H⋯H contacts in this study (51.4%). The graph shown in Fig. 6 ▸ *b* (H⋯C/C⋯H) shows the contacts between the carbon atoms inside the surface and the hydrogen atoms outside the surface of Hirshfeld and *vice versa* with two symmetrical wings on the left and right sides (26.7%). Two symmetrical points at the top, bottom left and right with *d*_e_ + *d*_i_ 2.5 Å indicate the presence of the H⋯C/C⋯H contacts. Further, there are H⋯O/O⋯H (8.9%), H⋯N/N⋯H (8%), C⋯C (3.2%) and C⋯O/O⋯C (1.0%) contacts.

**Figure 7:**
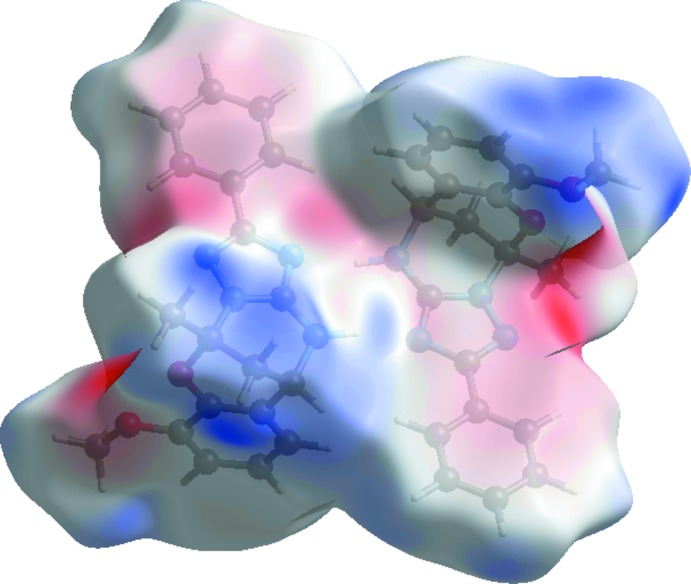
The view of the three-dimensional Hirshfeld surface of the title compound plotted over the electrostatic potential energy.

The view of the three-dimensional Hirshfeld surface of the title compound plotted over the electrostatic potential energy in the range −0.083 to 0.046 a.u. using the STO-3G basis set at the Hartree–Fock level of theory is shown in Fig. 7 ▸. The donors and acceptors are shown as blue and red areas around the atoms related with positive (hydrogen-bond donors) and negative (hydrogen-bond acceptors) electrostatic potentials, respectively.

## Database survey   

There are no direct precedents for the structure of the title compound in the crystallographic literature (CSD version 5.39; Groom *et al.*, 2016 ▸). However, there are several precedents for triazolobenzoxa­diazo­cines including 5-(2-hy­droxy­phen­yl)-7-methyl-4,5,6,7-tetra­hydro­[1,2,4]triazolo[1,5-*a*]pyrimidin-7-ol (Gorobets *et al.*, 2010 ▸), ethyl 7-chloro­methyl-5-(2-chloro­phen­yl)-7-hy­droxy-2-methyl­sulfanyl-4,5,6,7-tetra­hydro-1,2,4-triazolo[1,5-*a*]pyrimidine-6-carboxyl­ate (Huang, 2009 ▸), methyl 5′-(2-hy­droxy­phen­yl)-5′,6′-di­hydro-4′*H*-spiro­[chromene-2,7′-[1,2,4]triazolo[1,5-*a*]pyrimidine]-3-carboxyl­ate (Kett­mann & Světlík, 2011 ▸), 7-eth­oxy-5-methyl-2-(pyridin-3-yl)-11,12-di­hydro-5,11-methano­[1,2,4]triazolo[1,5-*c*][1,3,5]benzoxa­diazo­cine (Aydemir *et al.*, 2018 ▸) and 7-meth­oxy-5-methyl-2-(pyridin-3-yl)-11,12-di­hydro-5,11-methano­[1,2,4]triazolo[1,5-*c*][1,3,5]benzoxa­diazo­cine (Gümüş *et al.*, 2018 ▸).

## Synthesis and crystallization   

**Figure 8:**
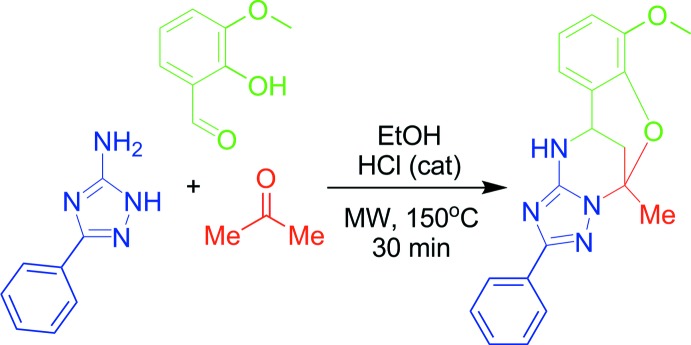
The synthesis of the title compound.

The synthesis (Fig. 8 ▸) of the title compound was described by Gümüş *et al.* (2017 ▸). 3-Amino-5-(phen­yl)-1,2,4-triazole (1.0 mmol), 2-hy­droxy-3-meth­oxy­benzaldehyde (1.0 mmol), acetone (0.22 mL, 3.0 mmol), and abs. EtOH (2.0 mL) were mixed in a microwave process vial, after which a 4 *N* solution of HCl in dioxane (0.07 mL, 0.3 mmol) was added. The mixture was irradiated at 423 K for 30 min. The reaction mixture was cooled by an air flow and stirred for 24 h at room temperature for complete precipitation of the product. The precipitate was filtered off, washed with EtOH (1.0 mL) and Et_2_O (3 × 1.0 mL), and dried. The compound was obtained in the form of a white solid with %53 yields. It was recrystallized from ethanol.

## Refinement   

Crystal data, data collection and structure refinement details are summarized in Table 2 ▸. The H atoms were positioned geometrically and refined using a riding model with N—H = 0.86 and C—H = 0.93–0.97 Å, *U*_iso_(H) = 1.2*U*_eq_(N,C).

## Supplementary Material

Crystal structure: contains datablock(s) I, global. DOI: 10.1107/S2056989018010848/xu5932sup1.cif

Structure factors: contains datablock(s) I. DOI: 10.1107/S2056989018010848/xu5932Isup2.hkl

Supporting information file. DOI: 10.1107/S2056989018010848/xu5932Isup3.cml

CCDC reference: 1852961

Additional supporting information:  crystallographic information; 3D view; checkCIF report

## Figures and Tables

**Figure 4:**
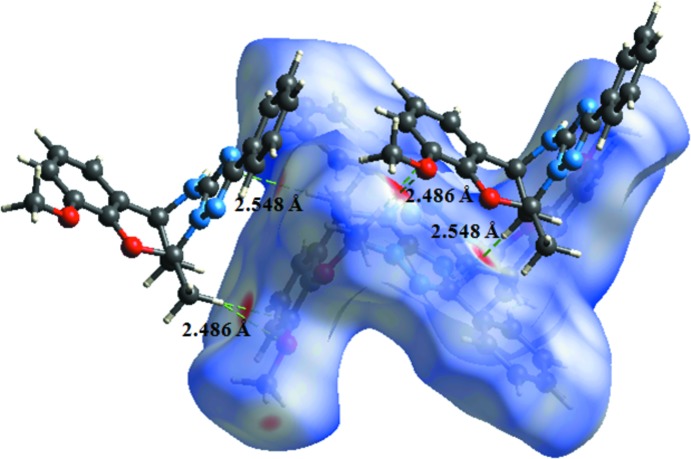
Hirshfeld surface mapped over *d*_norm_ for visualizing the inter­molecular inter­actions of the title compound.

**Table 1:** Hydrogen-bond geometry (Å, °) *Cg*1 is the centroid of the N1/C7/N2/N3/C8 ring.

*D*—H⋯*A*	*D*—H	H⋯*A*	*D*⋯*A*	*D*—H⋯*A*
N4—H4⋯N5	0.86	2.18	2.958 (3)	150
N8—H8⋯N1	0.86	2.33	3.025 (2)	139
C31—H31*A*⋯O4^i^	0.96	2.59	3.471 (3)	152
C38—H38*A*⋯O1^ii^	0.96	2.56	3.489 (3)	162
C12—H12*A*⋯*Cg*1^iii^	0.96	2.67	3.613 (3)	172

Symmetry codes: (i) 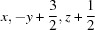; (ii) 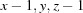; (iii) 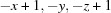.

**Table 2:** Experimental details

Crystal data	
Chemical formula	C_19_H_18_N_4_O_2_
*M*_r_	334.37
Crystal system, space group	Monoclinic, *P*2_1_/*c*
Temperature (K)	296
*a*, *b*, *c* (Å)	13.9787 (9), 21.5654 (12), 11.6625 (8)
β (°)	111.639 (5)
*V* (Å^3^)	3268.0 (4)
*Z*	8
Radiation type	Mo *K*α
μ (mm^−1^)	0.09
Crystal size (mm)	0.57 × 0.43 × 0.30

Data collection	
Diffractometer	Stoe IPDS 2
Absorption correction	Integration (*X-RED32*; Stoe & Cie, 2002 ▸)
*T*_min_, *T*_max_	0.959, 0.988
No. of measured, independent and observed [*I* > 2σ(*I*)] reflections	18118, 5769, 3357
*R*_int_	0.060
(sin θ/λ)_max_ (Å^−1^)	0.596

Refinement	
*R*[*F* ^2^ > 2σ(*F* ^2^)], *wR*(*F* ^2^), *S*	0.045, 0.104, 0.91
No. of reflections	5769
No. of parameters	451
H-atom treatment	H-atom parameters constrained
Δρ_max_, Δρ_min_ (e Å^−3^)	0.29, −0.25

Computer programs: *X-AREA* and *X-RED32* (Stoe & Cie, 2002 ▸), *ORTEP-3 for Windows* and *WinGX* (Farrugia, 1999 ▸), *SHELXL2017* (Sheldrick, 2015 ▸) and *PLATON* (Spek, 2009 ▸).

## supplementary crystallographic information

### Crystal data

**Table:**

C_19_H_18_N_4_O_2_	*F*(000) = 1408
*M**_r_* = 334.37	*D*_x_ = 1.359 Mg m^−^^3^
Monoclinic, *P*2_1_/*c*	Mo *K*α radiation, λ = 0.71073 Å
*a* = 13.9787 (9) Å	Cell parameters from 15386 reflections
*b* = 21.5654 (12) Å	θ = 1.8–27.5°
*c* = 11.6625 (8) Å	µ = 0.09 mm^−^^1^
β = 111.639 (5)°	*T* = 296 K
*V* = 3268.0 (4) Å^3^	Prism, yellow
*Z* = 8	0.57 × 0.43 × 0.30 mm

### Data collection

**Table:**

Stoe IPDS 2 diffractometer	5769 independent reflections
Radiation source: sealed X-ray tube, 12 x 0.4 mm long-fine focus	3357 reflections with *I* > 2σ(*I*)
Detector resolution: 6.67 pixels mm^-1^	*R*_int_ = 0.060
rotation method scans	θ_max_ = 25.1°, θ_min_ = 1.8°
Absorption correction: integration (X-RED32; Stoe & Cie, 2002)	*h* = −16→16
*T*_min_ = 0.959, *T*_max_ = 0.988	*k* = −25→25
18118 measured reflections	*l* = −13→13

### Refinement

**Table:**

Refinement on *F*^2^	0 restraints
Least-squares matrix: full	Hydrogen site location: inferred from neighbouring sites
*R*[*F*^2^ > 2σ(*F*^2^)] = 0.045	H-atom parameters constrained
*wR*(*F*^2^) = 0.104	*w* = 1/[σ^2^(*F*_o_^2^) + (0.047*P*)^2^] where *P* = (*F*_o_^2^ + 2*F*_c_^2^)/3
*S* = 0.91	(Δ/σ)_max_ < 0.001
5769 reflections	Δρ_max_ = 0.29 e Å^−^^3^
451 parameters	Δρ_min_ = −0.25 e Å^−^^3^

### Special details

**Table:**

Geometry. All esds (except the esd in the dihedral angle between two l.s. planes) are estimated using the full covariance matrix. The cell esds are taken into account individually in the estimation of esds in distances, angles and torsion angles; correlations between esds in cell parameters are only used when they are defined by crystal symmetry. An approximate (isotropic) treatment of cell esds is used for estimating esds involving l.s. planes.

### Fractional atomic coordinates and isotropic or equivalent isotropic displacement parameters (Å^2^)

**Table:**

	*x*	*y*	*z*	*U*_iso_*/*U*_eq_	
O1	0.57191 (11)	0.54141 (7)	0.85374 (13)	0.0513 (4)	
O3	−0.05290 (11)	0.70675 (7)	0.16610 (13)	0.0504 (4)	
O4	−0.12345 (12)	0.62171 (7)	0.00529 (15)	0.0609 (4)	
O2	0.65831 (13)	0.60242 (8)	1.05519 (15)	0.0693 (5)	
N2	0.56434 (13)	0.59962 (8)	0.60236 (17)	0.0476 (4)	
N7	0.02141 (13)	0.69913 (8)	0.38048 (16)	0.0480 (4)	
N3	0.49670 (13)	0.56839 (8)	0.64341 (16)	0.0466 (4)	
N5	0.12284 (13)	0.63678 (8)	0.52233 (16)	0.0480 (5)	
N1	0.40245 (13)	0.63875 (8)	0.51364 (16)	0.0458 (4)	
N6	−0.04391 (14)	0.66487 (8)	0.41952 (17)	0.0495 (5)	
N8	0.19756 (13)	0.70519 (8)	0.41385 (16)	0.0515 (5)	
H8	0.260678	0.704239	0.463886	0.062*	
N4	0.32207 (14)	0.57046 (9)	0.61530 (17)	0.0558 (5)	
H4	0.259042	0.578111	0.569932	0.067*	
C8	0.40190 (16)	0.59300 (10)	0.58996 (19)	0.0451 (5)	
C7	0.50387 (17)	0.64037 (10)	0.52610 (19)	0.0448 (5)	
C27	0.11950 (16)	0.68042 (10)	0.4408 (2)	0.0458 (5)	
C32	0.01999 (16)	0.67411 (10)	0.13675 (19)	0.0456 (5)	
C26	0.02015 (16)	0.62878 (10)	0.5035 (2)	0.0451 (5)	
C33	0.12485 (16)	0.68567 (10)	0.19011 (19)	0.0467 (5)	
C37	−0.01936 (17)	0.62810 (10)	0.0473 (2)	0.0483 (5)	
C6	0.54385 (17)	0.68468 (10)	0.4582 (2)	0.0474 (5)	
C13	0.50606 (17)	0.57244 (10)	0.8977 (2)	0.0492 (5)	
C28	0.16447 (17)	0.73388 (10)	0.2910 (2)	0.0503 (6)	
H28	0.222322	0.756287	0.282106	0.060*	
C25	−0.01654 (18)	0.58212 (10)	0.5693 (2)	0.0494 (6)	
C11	0.52605 (17)	0.51710 (10)	0.7309 (2)	0.0471 (5)	
C30	−0.01415 (17)	0.74234 (10)	0.2777 (2)	0.0483 (5)	
C9	0.35099 (17)	0.53199 (11)	0.7259 (2)	0.0555 (6)	
H9	0.289752	0.511153	0.729201	0.067*	
C14	0.40010 (18)	0.57065 (11)	0.8413 (2)	0.0534 (6)	
C34	0.19025 (18)	0.65033 (11)	0.1511 (2)	0.0548 (6)	
H34	0.260894	0.657001	0.185676	0.066*	
C29	0.07890 (17)	0.77909 (10)	0.2817 (2)	0.0543 (6)	
H29A	0.100512	0.806638	0.352464	0.065*	
H29B	0.062320	0.803995	0.207645	0.065*	
C18	0.55385 (19)	0.60704 (11)	1.0052 (2)	0.0556 (6)	
C36	0.04709 (19)	0.59434 (11)	0.0098 (2)	0.0567 (6)	
H36	0.021854	0.563977	−0.050559	0.068*	
C12	0.60649 (19)	0.47693 (11)	0.7120 (2)	0.0605 (6)	
H12A	0.579701	0.459349	0.630639	0.091*	
H12B	0.666141	0.501476	0.721056	0.091*	
H12C	0.625136	0.444240	0.772071	0.091*	
C10	0.42693 (18)	0.48407 (10)	0.7174 (2)	0.0574 (6)	
H10A	0.399673	0.462980	0.638432	0.069*	
H10B	0.439491	0.453458	0.782246	0.069*	
C35	0.15170 (19)	0.60569 (11)	0.0620 (2)	0.0588 (6)	
H35	0.196558	0.582758	0.036374	0.071*	
C5	0.64853 (19)	0.69075 (12)	0.4875 (2)	0.0670 (7)	
H5A	0.693835	0.666194	0.549337	0.080*	
C1	0.4784 (2)	0.72152 (11)	0.3660 (2)	0.0609 (6)	
H1	0.407676	0.718224	0.345098	0.073*	
C31	−0.10430 (18)	0.77995 (11)	0.2792 (2)	0.0613 (6)	
H31A	−0.083448	0.805098	0.352206	0.092*	
H31B	−0.128321	0.806149	0.207610	0.092*	
H31C	−0.158667	0.752615	0.278616	0.092*	
C20	0.0495 (2)	0.54167 (12)	0.6515 (2)	0.0645 (7)	
H20	0.119801	0.544843	0.668764	0.077*	
C24	−0.12103 (19)	0.57611 (12)	0.5459 (2)	0.0637 (7)	
H24	−0.167586	0.602879	0.490434	0.076*	
C15	0.3415 (2)	0.60652 (13)	0.8898 (3)	0.0688 (7)	
H15	0.270132	0.606084	0.852524	0.083*	
C17	0.4942 (2)	0.64257 (12)	1.0508 (2)	0.0676 (7)	
H17	0.525066	0.666583	1.121105	0.081*	
C38	−0.1696 (2)	0.57518 (13)	−0.0835 (3)	0.0798 (8)	
H38A	−0.242770	0.575837	−0.104823	0.120*	
H38B	−0.154160	0.582802	−0.155971	0.120*	
H38C	−0.143044	0.535338	−0.050002	0.120*	
C23	−0.1563 (2)	0.53131 (14)	0.6036 (3)	0.0762 (8)	
H23	−0.226406	0.528106	0.587450	0.091*	
C16	0.3881 (2)	0.64250 (13)	0.9919 (3)	0.0750 (8)	
H16	0.348124	0.667168	1.022098	0.090*	
C4	0.6866 (2)	0.73270 (14)	0.4262 (3)	0.0808 (8)	
H4A	0.757287	0.736288	0.447000	0.097*	
C19	0.7121 (2)	0.64069 (14)	1.1580 (3)	0.0842 (9)	
H19A	0.784592	0.632894	1.184031	0.126*	
H19B	0.698488	0.683463	1.134921	0.126*	
H19C	0.689387	0.631569	1.224482	0.126*	
C22	−0.0888 (3)	0.49106 (13)	0.6851 (3)	0.0756 (8)	
H22	−0.113075	0.460398	0.723457	0.091*	
C2	0.5172 (2)	0.76332 (12)	0.3045 (3)	0.0734 (8)	
H2	0.472295	0.787680	0.241927	0.088*	
C3	0.6211 (3)	0.76918 (13)	0.3349 (3)	0.0758 (8)	
H3	0.647028	0.797672	0.293850	0.091*	
C21	0.0138 (2)	0.49632 (12)	0.7093 (3)	0.0753 (8)	
H21	0.059945	0.469357	0.764810	0.090*	

### Atomic displacement parameters (Å^2^)

**Table:**

	*U*^11^	*U*^22^	*U*^33^	*U*^12^	*U*^13^	*U*^23^
O1	0.0424 (9)	0.0642 (9)	0.0441 (9)	0.0040 (7)	0.0120 (7)	−0.0015 (8)
O3	0.0413 (9)	0.0561 (9)	0.0489 (9)	0.0062 (7)	0.0109 (8)	−0.0038 (8)
O4	0.0468 (10)	0.0702 (10)	0.0618 (10)	−0.0085 (8)	0.0152 (8)	−0.0175 (9)
O2	0.0586 (11)	0.0860 (12)	0.0571 (11)	−0.0089 (9)	0.0139 (9)	−0.0156 (9)
N2	0.0389 (10)	0.0547 (11)	0.0478 (11)	−0.0005 (9)	0.0144 (9)	0.0001 (9)
N7	0.0377 (10)	0.0567 (11)	0.0464 (11)	0.0050 (9)	0.0119 (9)	0.0040 (9)
N3	0.0371 (10)	0.0518 (10)	0.0480 (11)	0.0030 (9)	0.0123 (9)	0.0039 (9)
N5	0.0426 (11)	0.0547 (11)	0.0447 (11)	0.0012 (9)	0.0137 (9)	0.0006 (9)
N1	0.0385 (11)	0.0512 (10)	0.0440 (10)	0.0010 (8)	0.0111 (9)	0.0011 (9)
N6	0.0414 (11)	0.0572 (11)	0.0507 (11)	0.0015 (9)	0.0179 (10)	−0.0004 (10)
N8	0.0368 (10)	0.0665 (12)	0.0450 (11)	−0.0043 (9)	0.0080 (9)	0.0059 (9)
N4	0.0343 (10)	0.0705 (12)	0.0549 (12)	−0.0032 (9)	0.0075 (9)	0.0133 (10)
C8	0.0379 (13)	0.0495 (12)	0.0428 (13)	0.0001 (10)	0.0091 (11)	−0.0018 (11)
C7	0.0417 (13)	0.0506 (13)	0.0399 (12)	−0.0005 (10)	0.0125 (11)	−0.0059 (11)
C27	0.0412 (13)	0.0512 (12)	0.0421 (12)	0.0017 (11)	0.0120 (11)	−0.0016 (11)
C32	0.0438 (13)	0.0502 (13)	0.0432 (13)	0.0048 (10)	0.0166 (11)	0.0055 (11)
C26	0.0412 (13)	0.0526 (13)	0.0411 (12)	0.0001 (11)	0.0146 (11)	−0.0043 (11)
C33	0.0442 (13)	0.0498 (12)	0.0460 (13)	0.0000 (10)	0.0164 (11)	0.0069 (11)
C37	0.0458 (14)	0.0527 (13)	0.0455 (13)	−0.0007 (11)	0.0158 (11)	0.0014 (11)
C6	0.0472 (14)	0.0494 (12)	0.0454 (13)	−0.0025 (11)	0.0169 (11)	−0.0059 (11)
C13	0.0490 (14)	0.0554 (13)	0.0456 (13)	0.0051 (11)	0.0203 (12)	0.0068 (11)
C28	0.0436 (13)	0.0534 (13)	0.0507 (14)	−0.0037 (11)	0.0137 (11)	0.0077 (11)
C25	0.0521 (14)	0.0550 (13)	0.0430 (13)	−0.0030 (11)	0.0200 (12)	−0.0063 (11)
C11	0.0438 (13)	0.0487 (12)	0.0430 (13)	0.0026 (10)	0.0090 (11)	0.0013 (11)
C30	0.0478 (13)	0.0473 (12)	0.0437 (13)	0.0055 (11)	0.0096 (11)	−0.0016 (11)
C9	0.0420 (14)	0.0609 (15)	0.0599 (15)	−0.0041 (11)	0.0144 (12)	0.0128 (12)
C14	0.0483 (14)	0.0624 (14)	0.0519 (14)	0.0024 (12)	0.0213 (12)	0.0121 (12)
C34	0.0430 (13)	0.0642 (15)	0.0592 (15)	0.0020 (11)	0.0213 (12)	0.0075 (13)
C29	0.0550 (15)	0.0459 (13)	0.0552 (14)	−0.0009 (11)	0.0123 (12)	0.0029 (11)
C18	0.0574 (16)	0.0635 (15)	0.0480 (14)	0.0015 (12)	0.0221 (13)	0.0029 (12)
C36	0.0606 (16)	0.0562 (14)	0.0560 (15)	0.0013 (12)	0.0246 (13)	−0.0027 (12)
C12	0.0595 (16)	0.0558 (14)	0.0598 (16)	0.0117 (12)	0.0146 (13)	−0.0031 (12)
C10	0.0533 (15)	0.0523 (13)	0.0578 (15)	−0.0061 (12)	0.0101 (12)	0.0056 (12)
C35	0.0576 (16)	0.0606 (15)	0.0672 (16)	0.0066 (13)	0.0334 (14)	−0.0001 (13)
C5	0.0531 (16)	0.0833 (18)	0.0658 (17)	−0.0021 (13)	0.0234 (14)	0.0112 (14)
C1	0.0578 (16)	0.0613 (15)	0.0627 (16)	0.0028 (12)	0.0211 (14)	0.0049 (13)
C31	0.0545 (15)	0.0592 (14)	0.0627 (16)	0.0116 (12)	0.0128 (13)	−0.0082 (12)
C20	0.0599 (16)	0.0727 (16)	0.0649 (16)	0.0024 (14)	0.0276 (14)	0.0071 (14)
C24	0.0527 (15)	0.0824 (17)	0.0585 (16)	−0.0047 (13)	0.0234 (13)	0.0025 (14)
C15	0.0570 (16)	0.0872 (19)	0.0704 (18)	0.0086 (15)	0.0331 (15)	0.0132 (16)
C17	0.081 (2)	0.0726 (17)	0.0567 (16)	−0.0002 (15)	0.0342 (16)	−0.0012 (13)
C38	0.0663 (18)	0.0786 (18)	0.089 (2)	−0.0166 (15)	0.0226 (16)	−0.0311 (17)
C23	0.0673 (19)	0.101 (2)	0.0685 (19)	−0.0221 (17)	0.0345 (16)	−0.0049 (17)
C16	0.079 (2)	0.0835 (19)	0.078 (2)	0.0133 (16)	0.0480 (18)	0.0033 (17)
C4	0.0661 (19)	0.100 (2)	0.085 (2)	−0.0130 (17)	0.0382 (18)	0.0117 (19)
C19	0.081 (2)	0.104 (2)	0.0644 (18)	−0.0348 (17)	0.0222 (16)	−0.0262 (17)
C22	0.097 (2)	0.0745 (18)	0.0685 (19)	−0.0187 (17)	0.0453 (19)	−0.0053 (15)
C2	0.090 (2)	0.0661 (16)	0.0674 (19)	0.0051 (15)	0.0335 (17)	0.0144 (14)
C3	0.093 (2)	0.0706 (17)	0.079 (2)	−0.0091 (17)	0.0497 (19)	0.0045 (16)
C21	0.095 (2)	0.0693 (17)	0.0699 (19)	0.0095 (16)	0.0395 (18)	0.0153 (15)

### Geometric parameters (Å, º)

**Table:**

O1—C13	1.380 (2)	C9—H9	0.9800
O1—C11	1.436 (3)	C14—C15	1.389 (3)
O3—C32	1.381 (2)	C34—C35	1.373 (3)
O3—C30	1.434 (3)	C34—H34	0.9300
O4—C37	1.360 (3)	C29—H29A	0.9700
O4—C38	1.415 (3)	C29—H29B	0.9700
O2—C18	1.362 (3)	C18—C17	1.376 (3)
O2—C19	1.421 (3)	C36—C35	1.383 (3)
N2—C7	1.313 (3)	C36—H36	0.9300
N2—N3	1.382 (2)	C12—H12A	0.9600
N7—C27	1.351 (3)	C12—H12B	0.9600
N7—N6	1.376 (2)	C12—H12C	0.9600
N7—C30	1.454 (3)	C10—H10A	0.9700
N3—C8	1.348 (3)	C10—H10B	0.9700
N3—C11	1.457 (3)	C35—H35	0.9300
N5—C27	1.326 (3)	C5—C4	1.376 (3)
N5—C26	1.381 (3)	C5—H5A	0.9300
N1—C8	1.331 (3)	C1—C2	1.381 (3)
N1—C7	1.371 (3)	C1—H1	0.9300
N6—C26	1.312 (3)	C31—H31A	0.9600
N8—C27	1.352 (3)	C31—H31B	0.9600
N8—C28	1.470 (3)	C31—H31C	0.9600
N8—H8	0.8600	C20—C21	1.381 (3)
N4—C8	1.346 (3)	C20—H20	0.9300
N4—C9	1.460 (3)	C24—C23	1.368 (3)
N4—H4	0.8600	C24—H24	0.9300
C7—C6	1.476 (3)	C15—C16	1.369 (4)
C32—C33	1.387 (3)	C15—H15	0.9300
C32—C37	1.397 (3)	C17—C16	1.386 (4)
C26—C25	1.468 (3)	C17—H17	0.9300
C33—C34	1.390 (3)	C38—H38A	0.9600
C33—C28	1.514 (3)	C38—H38B	0.9600
C37—C36	1.373 (3)	C38—H38C	0.9600
C6—C1	1.378 (3)	C23—C22	1.373 (4)
C6—C5	1.381 (3)	C23—H23	0.9300
C13—C14	1.382 (3)	C16—H16	0.9300
C13—C18	1.399 (3)	C4—C3	1.368 (4)
C28—C29	1.516 (3)	C4—H4A	0.9300
C28—H28	0.9800	C19—H19A	0.9600
C25—C20	1.371 (3)	C19—H19B	0.9600
C25—C24	1.390 (3)	C19—H19C	0.9600
C11—C12	1.499 (3)	C22—C21	1.361 (4)
C11—C10	1.514 (3)	C22—H22	0.9300
C30—C31	1.504 (3)	C2—C3	1.369 (4)
C30—C29	1.509 (3)	C2—H2	0.9300
C9—C10	1.511 (3)	C3—H3	0.9300
C9—C14	1.515 (3)	C21—H21	0.9300
			
C13—O1—C11	115.72 (17)	C28—C29—H29A	110.1
C32—O3—C30	115.55 (16)	C30—C29—H29B	110.1
C37—O4—C38	118.28 (19)	C28—C29—H29B	110.1
C18—O2—C19	118.0 (2)	H29A—C29—H29B	108.4
C7—N2—N3	102.06 (16)	O2—C18—C17	125.6 (2)
C27—N7—N6	109.69 (17)	O2—C18—C13	115.3 (2)
C27—N7—C30	126.41 (18)	C17—C18—C13	119.1 (2)
N6—N7—C30	123.39 (17)	C37—C36—C35	119.7 (2)
C8—N3—N2	109.23 (17)	C37—C36—H36	120.1
C8—N3—C11	126.70 (18)	C35—C36—H36	120.1
N2—N3—C11	124.07 (17)	C11—C12—H12A	109.5
C27—N5—C26	102.41 (18)	C11—C12—H12B	109.5
C8—N1—C7	102.05 (17)	H12A—C12—H12B	109.5
C26—N6—N7	102.24 (17)	C11—C12—H12C	109.5
C27—N8—C28	113.71 (18)	H12A—C12—H12C	109.5
C27—N8—H8	123.1	H12B—C12—H12C	109.5
C28—N8—H8	123.1	C9—C10—C11	108.04 (18)
C8—N4—C9	114.67 (18)	C9—C10—H10A	110.1
C8—N4—H4	122.7	C11—C10—H10A	110.1
C9—N4—H4	122.7	C9—C10—H10B	110.1
N1—C8—N4	128.6 (2)	C11—C10—H10B	110.1
N1—C8—N3	110.74 (18)	H10A—C10—H10B	108.4
N4—C8—N3	120.62 (19)	C34—C35—C36	120.9 (2)
N2—C7—N1	115.91 (19)	C34—C35—H35	119.5
N2—C7—C6	121.35 (19)	C36—C35—H35	119.5
N1—C7—C6	122.74 (19)	C4—C5—C6	120.8 (3)
N5—C27—N7	110.24 (18)	C4—C5—H5A	119.6
N5—C27—N8	129.0 (2)	C6—C5—H5A	119.6
N7—C27—N8	120.78 (19)	C6—C1—C2	120.5 (2)
O3—C32—C33	123.44 (19)	C6—C1—H1	119.8
O3—C32—C37	115.06 (19)	C2—C1—H1	119.8
C33—C32—C37	121.50 (19)	C30—C31—H31A	109.5
N6—C26—N5	115.36 (19)	C30—C31—H31B	109.5
N6—C26—C25	121.54 (19)	H31A—C31—H31B	109.5
N5—C26—C25	123.1 (2)	C30—C31—H31C	109.5
C32—C33—C34	117.9 (2)	H31A—C31—H31C	109.5
C32—C33—C28	119.68 (19)	H31B—C31—H31C	109.5
C34—C33—C28	122.3 (2)	C25—C20—C21	121.4 (2)
O4—C37—C36	126.2 (2)	C25—C20—H20	119.3
O4—C37—C32	114.56 (19)	C21—C20—H20	119.3
C36—C37—C32	119.2 (2)	C23—C24—C25	120.8 (3)
C1—C6—C5	118.4 (2)	C23—C24—H24	119.6
C1—C6—C7	121.2 (2)	C25—C24—H24	119.6
C5—C6—C7	120.3 (2)	C16—C15—C14	120.5 (3)
O1—C13—C14	123.7 (2)	C16—C15—H15	119.8
O1—C13—C18	115.3 (2)	C14—C15—H15	119.8
C14—C13—C18	121.0 (2)	C18—C17—C16	119.8 (3)
N8—C28—C33	111.22 (17)	C18—C17—H17	120.1
N8—C28—C29	107.28 (18)	C16—C17—H17	120.1
C33—C28—C29	109.47 (19)	O4—C38—H38A	109.5
N8—C28—H28	109.6	O4—C38—H38B	109.5
C33—C28—H28	109.6	H38A—C38—H38B	109.5
C29—C28—H28	109.6	O4—C38—H38C	109.5
C20—C25—C24	117.7 (2)	H38A—C38—H38C	109.5
C20—C25—C26	121.8 (2)	H38B—C38—H38C	109.5
C24—C25—C26	120.4 (2)	C24—C23—C22	120.4 (3)
O1—C11—N3	109.16 (16)	C24—C23—H23	119.8
O1—C11—C12	105.96 (18)	C22—C23—H23	119.8
N3—C11—C12	111.61 (18)	C15—C16—C17	120.7 (3)
O1—C11—C10	109.30 (18)	C15—C16—H16	119.6
N3—C11—C10	105.88 (18)	C17—C16—H16	119.6
C12—C11—C10	114.85 (18)	C3—C4—C5	120.5 (3)
O3—C30—N7	107.77 (16)	C3—C4—H4A	119.8
O3—C30—C31	105.15 (18)	C5—C4—H4A	119.8
N7—C30—C31	111.67 (18)	O2—C19—H19A	109.5
O3—C30—C29	110.02 (18)	O2—C19—H19B	109.5
N7—C30—C29	106.40 (18)	H19A—C19—H19B	109.5
C31—C30—C29	115.63 (18)	O2—C19—H19C	109.5
N4—C9—C10	107.63 (19)	H19A—C19—H19C	109.5
N4—C9—C14	111.05 (18)	H19B—C19—H19C	109.5
C10—C9—C14	109.61 (19)	C21—C22—C23	119.6 (3)
N4—C9—H9	109.5	C21—C22—H22	120.2
C10—C9—H9	109.5	C23—C22—H22	120.2
C14—C9—H9	109.5	C3—C2—C1	120.6 (3)
C13—C14—C15	118.7 (2)	C3—C2—H2	119.7
C13—C14—C9	119.5 (2)	C1—C2—H2	119.7
C15—C14—C9	121.7 (2)	C4—C3—C2	119.3 (3)
C35—C34—C33	120.7 (2)	C4—C3—H3	120.3
C35—C34—H34	119.6	C2—C3—H3	120.3
C33—C34—H34	119.6	C22—C21—C20	120.0 (3)
C30—C29—C28	108.22 (17)	C22—C21—H21	120.0
C30—C29—H29A	110.1	C20—C21—H21	120.0
			
C7—N2—N3—C8	−0.6 (2)	N2—N3—C11—C10	−161.78 (18)
C7—N2—N3—C11	179.45 (18)	C32—O3—C30—N7	−68.6 (2)
C27—N7—N6—C26	1.6 (2)	C32—O3—C30—C31	172.12 (17)
C30—N7—N6—C26	173.82 (18)	C32—O3—C30—C29	47.0 (2)
C7—N1—C8—N4	−179.8 (2)	C27—N7—C30—O3	97.7 (2)
C7—N1—C8—N3	−0.7 (2)	N6—N7—C30—O3	−73.2 (2)
C9—N4—C8—N1	−164.4 (2)	C27—N7—C30—C31	−147.3 (2)
C9—N4—C8—N3	16.5 (3)	N6—N7—C30—C31	41.8 (3)
N2—N3—C8—N1	0.9 (2)	C27—N7—C30—C29	−20.3 (3)
C11—N3—C8—N1	−179.18 (17)	N6—N7—C30—C29	168.82 (18)
N2—N3—C8—N4	−179.95 (18)	C8—N4—C9—C10	−50.3 (2)
C11—N3—C8—N4	0.0 (3)	C8—N4—C9—C14	69.7 (2)
N3—N2—C7—N1	0.1 (2)	O1—C13—C14—C15	−176.4 (2)
N3—N2—C7—C6	−179.56 (18)	C18—C13—C14—C15	3.3 (3)
C8—N1—C7—N2	0.3 (2)	O1—C13—C14—C9	1.1 (3)
C8—N1—C7—C6	−179.96 (19)	C18—C13—C14—C9	−179.18 (19)
C26—N5—C27—N7	2.3 (2)	N4—C9—C14—C13	−99.0 (2)
C26—N5—C27—N8	−176.9 (2)	C10—C9—C14—C13	19.8 (3)
N6—N7—C27—N5	−2.6 (2)	N4—C9—C14—C15	78.5 (3)
C30—N7—C27—N5	−174.52 (18)	C10—C9—C14—C15	−162.7 (2)
N6—N7—C27—N8	176.73 (18)	C32—C33—C34—C35	0.3 (3)
C30—N7—C27—N8	4.8 (3)	C28—C33—C34—C35	178.0 (2)
C28—N8—C27—N5	157.9 (2)	O3—C30—C29—C28	−66.0 (2)
C28—N8—C27—N7	−21.2 (3)	N7—C30—C29—C28	50.4 (2)
C30—O3—C32—C33	−15.9 (3)	C31—C30—C29—C28	175.05 (19)
C30—O3—C32—C37	164.59 (18)	N8—C28—C29—C30	−69.0 (2)
N7—N6—C26—N5	−0.1 (2)	C33—C28—C29—C30	51.8 (2)
N7—N6—C26—C25	−178.34 (18)	C19—O2—C18—C17	−4.9 (4)
C27—N5—C26—N6	−1.4 (2)	C19—O2—C18—C13	174.5 (2)
C27—N5—C26—C25	176.84 (19)	O1—C13—C18—O2	−3.6 (3)
O3—C32—C33—C34	−178.85 (19)	C14—C13—C18—O2	176.7 (2)
C37—C32—C33—C34	0.6 (3)	O1—C13—C18—C17	175.9 (2)
O3—C32—C33—C28	3.5 (3)	C14—C13—C18—C17	−3.8 (3)
C37—C32—C33—C28	−177.07 (19)	O4—C37—C36—C35	−179.4 (2)
C38—O4—C37—C36	1.7 (3)	C32—C37—C36—C35	0.9 (3)
C38—O4—C37—C32	−178.6 (2)	N4—C9—C10—C11	69.0 (2)
O3—C32—C37—O4	−1.5 (3)	C14—C9—C10—C11	−51.9 (2)
C33—C32—C37—O4	179.01 (19)	O1—C11—C10—C9	66.9 (2)
O3—C32—C37—C36	178.25 (19)	N3—C11—C10—C9	−50.6 (2)
C33—C32—C37—C36	−1.3 (3)	C12—C11—C10—C9	−174.19 (19)
N2—C7—C6—C1	171.0 (2)	C33—C34—C35—C36	−0.6 (4)
N1—C7—C6—C1	−8.7 (3)	C37—C36—C35—C34	0.0 (4)
N2—C7—C6—C5	−10.1 (3)	C1—C6—C5—C4	0.1 (4)
N1—C7—C6—C5	170.2 (2)	C7—C6—C5—C4	−178.8 (2)
C11—O1—C13—C14	12.7 (3)	C5—C6—C1—C2	0.2 (4)
C11—O1—C13—C18	−166.98 (18)	C7—C6—C1—C2	179.1 (2)
C27—N8—C28—C33	−67.2 (2)	C24—C25—C20—C21	0.1 (4)
C27—N8—C28—C29	52.5 (2)	C26—C25—C20—C21	−177.4 (2)
C32—C33—C28—N8	95.9 (2)	C20—C25—C24—C23	0.1 (3)
C34—C33—C28—N8	−81.7 (2)	C26—C25—C24—C23	177.7 (2)
C32—C33—C28—C29	−22.5 (3)	C13—C14—C15—C16	−0.5 (4)
C34—C33—C28—C29	159.9 (2)	C9—C14—C15—C16	−178.0 (2)
N6—C26—C25—C20	175.8 (2)	O2—C18—C17—C16	−179.1 (2)
N5—C26—C25—C20	−2.2 (3)	C13—C18—C17—C16	1.5 (4)
N6—C26—C25—C24	−1.6 (3)	C25—C24—C23—C22	−0.5 (4)
N5—C26—C25—C24	−179.7 (2)	C14—C15—C16—C17	−1.8 (4)
C13—O1—C11—N3	69.0 (2)	C18—C17—C16—C15	1.2 (4)
C13—O1—C11—C12	−170.65 (17)	C6—C5—C4—C3	−0.1 (4)
C13—O1—C11—C10	−46.4 (2)	C24—C23—C22—C21	0.7 (4)
C8—N3—C11—O1	−99.3 (2)	C6—C1—C2—C3	−0.6 (4)
N2—N3—C11—O1	80.7 (2)	C5—C4—C3—C2	−0.3 (4)
C8—N3—C11—C12	143.9 (2)	C1—C2—C3—C4	0.7 (4)
N2—N3—C11—C12	−36.1 (3)	C23—C22—C21—C20	−0.4 (4)
C8—N3—C11—C10	18.3 (3)	C25—C20—C21—C22	0.0 (4)

### Hydrogen-bond geometry (Å, º)

Cg1 is the centroid of the N1/C7/N2/N3/C8 ring.

**Table:**

*D*—H···*A*	*D*—H	H···*A*	*D*···*A*	*D*—H···*A*
N4—H4···N5	0.86	2.18	2.958 (3)	150
N8—H8···N1	0.86	2.33	3.025 (2)	139
C31—H31*A*···O4^i^	0.96	2.59	3.471 (3)	152
C38—H38*A*···O1^ii^	0.96	2.56	3.489 (3)	162
C12—H12*A*···*Cg*1^iii^	0.96	2.67	3.613 (3)	172

Symmetry codes: (i) *x*, −*y*+3/2, *z*+1/2; (ii) *x*−1, *y*, *z*−1; (iii) −*x*+1, −*y*, −*z*+1.
